# Research on the innovation of time-honored brands from the perspective of dual ethical patterns

**DOI:** 10.3389/fpsyg.2022.1041022

**Published:** 2023-01-31

**Authors:** Di Ke, Guodong Li, Yichen Jiang, Yuanyuan Li, Yi Liu

**Affiliations:** ^1^School of Economics and Management, Civil Aviation University of China, Tianjin, China; ^2^Zhejiang Science and Technology Information Research Institute, Hangzhou, China; ^3^School of Business Administration, Shanxi University of Finance and Economics, Taiyuan, Shanxi, China; ^4^School of Economics and Management, Tianjin Agricultural University, Tianjin, China

**Keywords:** time-honored brands, traditional culture, marketization level, enterprise innovation, fuzzy set qualitative comparative analysis

## Abstract

**Introduction:**

Innovation has become an important means to promote the high-quality development of time-honored brands. However, the research on how to stimulate innovation investment in time-honored brands, especially that conducted in the local context, is still rare. To supplement the research limitations, this study adopts the perspective of dual ethical patterns and is set in the domestic context to explore the ethical influence of traditional cultures and marketization on the innovation investment of time-honored brands.

**Methods:**

We proposed two complementary methods, which are OLS regression and fsQCA analysis respectively, to systematically analyze the mechanism and key path of the dual ethical pattern to promote the innovation of time-honored brands.

**Results:**

The results show that traditional culture and marketization level are both positively related to the innovation of time-honored brands. However, traditional culture and marketization level are mutually exclusive in their process of affecting the innovation of time-honored brands.

**Discussion:**

This paper advances time-honored brands literature by highlighting the dual ethic pattern formed by traditional culture and marketization level on the innovation investment of time-honored brands. The findings respond to the academic debate between traditional culture and the innovation of time-honored brands, while enriching the research scope on the innovation mechanism of time-honored brands in the local context.

## Introduction

Time-honored brands, as an important part of national brands, are crucial to driving the development of China’s brand economy (Zhang et al., 2021). However, due to various reasons, the once-thriving time-honored brands are now facing a survival crisis and development difficulties as it goes through a sharp decline in all aspects (Li et al., 2019). One important reason for this situation is that most of the time-honored brands are lagging behind in technological research and development, lack of innovation, and cannot keep up with the changes in the consumer environment (Wang et al., 2022). That innovation is crucial to boosting the revitalization and sustainable development of enterprises (Saura et al., 2021, 2022; Yi et al., 2022; Zhao, 2022), especially for time-honored brands, has been widely recognized in academia and practical circles (Wang and Wang, 2020; Saci et al., 2021; Wang et al., 2022).

According to institutional theory, a firm’s decision-making behavior is shaped by its external institutional environment (Meyer and Rowan, 1977; Pan et al., 2022). In China, the representative institutional environment variables are traditional culture and the level of marketization (Xia et al., 2020). Since China’s reform and opening up, changes in the institutional environment have placed China in a dual-ethical pattern of traditional culture and marketization, with real and unique Chinese situational elements (Wang et al., 2016). This dual ethical pattern has had a huge influence on the business decisions of Chinese enterprises.

On the one hand, traditional culture, with traditional ethical value systems such as benevolence, righteousness, manners, wisdom, and credit as its core, plays an important role in the operation of time-honored brands (Wang and Juslin, 2009; Kung and Ma, 2014); on the other hand, since 1978, with the historic transition from a traditional planned economy to a market economy, time-honored brands have also been influenced by the market-oriented ethics of competition, self-interest, and equitable exchange. At present, there is no consensus in academic circles on whether the two ethical patterns of traditional culture and marketization coexist harmoniously or conflict with each other (Gu et al., 2008; Zhou et al., 2014).

Time-honored brands, as the unique local enterprises deeply rooted in traditional Chinese culture and developed under market-oriented economic reforms (Balmer and Chen, 2017), are more likely to be influenced by the dual ethical patterns. Therefore, it is essential to study the impact of the dual ethical patterns of traditional culture and marketization on the technological innovation behavior of time-honored brands. However, the existing research on the key factors affecting the innovation of time-honored brands is restricted to the perspectives of market demand (Bowonder et al., 2010; Huang, 2022), government policies (Li and Yang, 2021), and corporate governance (Shang et al., 2021), with low authority and little impact. More regrettably, few scholars have paid attention to the specific influencing factors of the local context on the innovation of time-honored brands.

In this context, the present study aims to investigate the link between the dual ethical patterns and the innovation of time-honored brands, which contributes to expanding the research paradigm of “culture and finance.” Moreover, we also explore the combination configuration effect of dual ethical patterns and mutual dependence and combination of enterprises’ own factors on the innovation of time-honored brands, which fills a gap in the literature by the analysis of innovation of time-honored brands both from the causality and configuration perspectives.

The research questions addressed in the present study are as follows:

RQ1: What is the influence of traditional culture on the innovation of time-honored brands?

RQ2: Does the difference in the level of marketization in various regions of China affect the innovation investment of time-honored brands of the locality?

RQ3: Are the two ethical patterns of traditional culture and marketization level complementary or mutually exclusive in the innovation of time-honored brands?

RQ4: How can the enterprise’s own situational variables be coordinated with the dual-ethical pattern to maximize the innovation level of time-honored brands?

In terms of methodology, the approach adopted in the present study unfolds in the following two steps. First, we use multiple regression analysis to verify the influence of traditional culture and marketization level on the technological innovation of time-honored brands. Second, based on its findings, we conduct an in-depth analysis with the fsQCA research method to explain the complex causal mechanism of traditional culture, marketization level, and relevant situational factors that jointly affect the innovation of time-honored brands. The reason we combine OLS and fsQCA analysis is that regression analysis can only analyze the interaction of dual ethical patterns on enterprise innovation from a linear perspective. However, the fsQCA method can further analyze the key path that triggers the innovation of time-honored brands from a non-linear perspective.

This study adds scientific value by revealing the impact of the dual ethical patterns on the innovation of time-honored brands in the local context, and explores the key path of enterprise innovation from many aspects, supplementing the relevant theories of institutional and enterprise innovation. To the best of our knowledge, this study is the first to combine institutional (marketization level) and informal institutional (traditional culture) perspectives to examine the impact of different institutional environments on the innovation of time-honored brands. Thus, this study not only deepens the understanding of the cultural soil on which innovative behavior rests and the logic of its power but also enriches the literature on enterprise innovation. In addition, previous studies have not reached a consensus on the harmony between market ethics and cultural ethics. This manuscript reveals the theoretical logic and empirical evidence of the influence of the two ethics on enterprise innovation and responds to the academic debate between traditional culture and the innovation of time-honored brands.

The remainder of this manuscript is structured as follows. In the section “Literature review and research hypotheses,” we review relevant literature and propose research hypotheses. The methodology is described in the section “Methodology”. Section “Empirical results” presents the OLS regression results. In the section “Extended analysis: a qualitative comparative analysis of fuzzy sets,” we introduce the fsQCA research method to explain the complex causal mechanism of traditional culture, marketization level, and relevant situational factors that jointly affect the innovation of time-honored brands. Section “Discussion” discusses these findings. Finally, conclusions, theoretical and practical implications, as well as current limitations are identified in the section “Conclusion.”

## Literature review and research hypotheses

### Traditional culture and the innovation of time-honored brands

The imprinting theory holds that entrepreneurs’ cognition and behavior will take on various characteristics after being influenced by their own culture (Marquis and Tilcsik, 2013; Yin et al., 2014). According to Wang et al. (2022), the values of Chinese entrepreneurs are permeated by the ideas of the traditional culture they receive and are reflected in their business decisions. Rooted in traditional Chinese culture, time-honored brands are a unique local phenomenon (Zhou et al., 2022). Compared with new-type companies, time-honored brands are more deeply influenced by the traditional culture due to their long history. Therefore, traditional culture has a more profound impact on the innovation behavior of time-honored companies. This manuscript will interpret the classic documents of traditional culture and the ideas and elaborations closely related to the innovation activities of time-honored brands. The details are as follows:

The positive and enterprising spirit of innovation contained in the core concept of traditional culture has stimulated the innovation of time-honored brands, mainly reflected in three aspects: First, in the context of the relatively weak intellectual property legal system in China (Keupp et al., 2010; Huang et al., 2017), the viewpoint on justice and interests and the focus on integrity in traditional culture, as the long-standing management methods and existence foundation for time-honored brands, are conductive to regulating the behavior of competitors and stimulating the innovation enthusiasm of time-honored brands. Second, traditional culture attaches great importance to knowledge, education, and talents (Tho, 2016), which are important resources for the technological innovation of time-honored brands. Value concepts such as “being insatiable in learning and tireless in teaching” (*The Analects of Confucius ⋅ Shu’er*) and “Is it not pleasant to learn with a constant perseverance and application?” (*The Analects of Confucius ⋅ Xue’er*) are the true portrayals of the respect for knowledge and talents of traditional culture. Respect for knowledge and talent is an essential prerequisite for the long-standing development of time-honored brands. The rich knowledge treasures (such as unique craftsmanship and recipes), and technical talents that master unique skills are the basic power for the time-honored brands to embark on innovative activities. Third, traditional culture has a pioneering and enterprising spirit and is always willing to seek self-improvement (Xu et al., 2019). The sentence, “As Heaven changes through movement, a gentleman makes unremitting efforts to perfect himself” (from the *Book of Changes*) is a good interpretation of the time-honored brand’s national spirit for constant self-improvement that has been inherited over thousands of years. Such a spiritual connotation is highly consistent with time-honored brands’ pursuit of technological innovation and the strong will to overcome difficulties. Therefore, it plays a crucial role in promoting the reform and innovation activities of time-honored brands.

Although the traditional culture possesses the natural endowment to promote the innovation of time-honored brands, there are also negative factors that hinder the innovation and improvement of time-honored brands, mainly embodied in the following aspects: First, traditional culture emphasizes more stability than development. The pursuit of stability is the theme of traditional cultural values. In *The Analects of Confucius, Yan Yuan*, Confucius emphasized the behavior to “restrain yourself and follow social norms,” requiring that people “do not see, listen, say, or act inappropriately.” The main aim of such ideas is to maintain the stability of the *status quo* and to clarify the social relationship. However, the innovation process is accompanied by conflicts of interest (Vollmer, 2015), and may even threaten organizational stability, thus strongly opposed by traditional culture. Many time-honored brands insist on operating only one store and resist opening branch stores, and blindly pursue stability rather than development, which hinders their innovative development. Second, “small wealth is a good wealth” is not only a common life attitude in traditional Chinese culture but also a typical traditional way of thinking. The well-known aphorism, such as “the modest receive benefit while the conceited reap failure” (from the *Book of History*), “contentment is not humiliating” (from *Lao Tzu*), and so on well explain the idea of “small wealth is a good wealth.” The belief that “small wealth is good wealth” is also a common profit concept for many time-honored companies. This way of thinking makes some time-honored brands feel comfortable with the *status quo* and unwilling to make progress, which results in their lack of innovative attitude. Third, traditional culture has a negative attitude toward fierce competition. Mencius emphasized that “the heart to give precedence to somebody else marks the start of courtesy” (from *Mengzi ⋅ Gongsun Chou I*). Confucius also emphasized that “it is good to give precedence to others and avoid competence.” With harmony as its core value (Fan, 2000; Murphy and Wang, 2006), traditional culture believes that it is necessary to actively adapt to the internal and external environment while avoiding conflicts. Some time-honored brands are still adhering to the adage that “the fragrance of wine is not afraid of deep alleys,” and have not responded to the fierce competition in the market. Such an outdated way of thinking may have a certain inhibitory effect on the innovative behavior of time-honored brands. It can be seen that a competitive hypothesis mechanism exists concerning the influence of traditional culture on the innovation of time-honored brand enterprises. To comprehensively analyze the systematic impact of traditional culture on the innovation of time-honored brands, this research proposes the following hypotheses from both positive and negative sides.

**Hypothesis 1a:** Traditional culture is conducive to promote the innovation of time-honored brands.

**Hypothesis 1b:** Traditional culture has an inhibitory effect on the innovation of time-honored brands.

### Marketization level and the innovation of time-honored brands

Innovation is relatively highly dependent on the development of the regional system (Oluwatobi et al., 2015; Silve and Plekhanov, 2018; He and Tian, 2020). Affected by geographical location, government policies, and other factors, the level of marketization in China is extremely imbalanced among different regions (Chan et al., 2010; Chang and Wu, 2014; Jia, 2014; Sheng et al., 2018; Piao and Moon, 2019). Time-honored brands are enterprises with obvious regional characteristics. Therefore, the innovation behavior of time-honored brands is closely related to the level of marketization of the local region. First of all, the market competition in areas with a high level of marketization is also more intense (Jia et al., 2021). To achieve development and revitalization, the time-honored brand must change the backward concept that “the fragrance of wine is not afraid of deep alleys” and be actively involved in market competition. A relatively strong market competition will effectively drive the innovation investment of enterprises (Norbäck and Persson, 2012; Zhou et al., 2014; Marshall and Parra, 2019; Parra, 2019). Secondly, the lack of effective protection of the proprietary technology and intellectual property rights is an essential factor hindering the innovative development of time-honored brands (Pisuke and Kelli, 2008; Hussain and Terziovski, 2016; Liu and Jiang, 2016). In areas with a relatively low level of marketization, the intellectual property rights of technological achievements cannot be effectively protected, which will reduce the innovation motivation of time-honored brands. However, in areas with a high level of marketization, the level of legalization is also relatively high (Klapper and Love, 2004). In addition to strengthening the protection of the intellectual property rights of enterprises, a sound and sophisticated legal system can also guarantee the overflow channels of technological innovation (Cai et al., 2016; He et al., 2018) and shorten the time for enterprise innovation. Finally, regions with a high level of marketization have a loose innovation policy environment, which is conducive to alleviating the policy pressure of the government. Moreover, the capital market in these regions is also relatively developed, thus providing policy and financial resource support for the innovation of time-honored brands.

Based on the analysis above, we propose the following assumptions:

**Hypothesis 2:** High marketization level can promote the innovation of time-honored brands.

### Traditional culture, marketization level, and the innovation of time-honored brands

Although time-honored brands are rooted in Chinese traditional culture, they are developed in the context of China’s reform of the market economy. Therefore, the fusion and collision of old and new forces will have an important impact on the innovation behavior of time-honored brands. Under such a background, the dual influence of traditional culture and marketization must be considered simultaneously when examining the innovation behavior of time-honored brands.

Only when traditional culture and marketization are compatible with one another can they have a supplementary effect on the innovation process of time-honored brands. First of all, to gain maximal profit and meet the ever-changing needs of consumers, time-honored brands must change the traditional production mode of manual workshops, and reduce costs by innovating the production technologies. In *The Analects of Confucius*, Confucius said that “wealth and fame are a common desire of human beings; however, those who do not properly gain wealth or fame do not worth getting along with.” The connotation of the sentence that wealth and fame are desired by everyone is in line with the advocation of the market economy for the enterprise to maximize their interests. In this regard, traditional culture can serve as a supplementary force for marketization. In this circumstance, time-honored brands lay greater emphasis on investing in technological innovation and further expanding their interests. Secondly, defects and failures inevitably occur during the operation of the market economy, which may seriously discourage enterprises from seeking innovation (Williams and Tsiteladze, 2019). As traditional Chinese culture gives high status to moral practice, it can to some extent make up for the series of problems caused by market failure. In addition, traditional culture can effectively alleviate the agency problem in the process of market operation (Saci et al., 2021). In this way, the opportunistic behavior of managers can be inhibited, and the managers will be forced to actively carry out innovative research and development activities that are conducive to increasing corporate value. To sum up, traditional beliefs and spiritual heritage have always been deeply rooted in time-honored brands and can serve as a supplementary force in a market-oriented economy. The integration of the two aspects is conducive to promoting the innovative development of time-honored brands.

However, traditional culture and the marketization level may also have mutually exclusive effects when they conflict with each other in the innovation process of time-honored brands. Firstly, traditional culture and marketization are mutually exclusive at a competitive level. With an emphasis on fairness and an objection to competition, traditional culture is not suitable for the development of a market economy. As is written in Chapter XVI of The Analects of Confucius, Ji Shi, “Inequality rather than want is the cause of trouble.” Such a philosophy of evenness runs in the opposite direction to competition. According to the traditional Chinese etiquette, “What you do not wish yourself, do not do unto others.” In other words, it is believed that competition is immoral. The emphasis on the golden mean and tolerance in traditional culture also stifles people’s sense of competition. However, competition is the product of marketization, which requires an innovative spirit (Grossman and Lai, 2004; Yang et al., 2021). The fierce competition forces enterprises to adopt new workmanship, apply new technologies, and produce new products in order to meet the ever-changing consumer demands (Lin et al., 2020). Secondly, the conceptual conflict between the rule of man and the rule of law can lead to the mutual exclusiveness between the traditional culture and marketization. The legal system is the basic feature of marketization (Yao and Yueh, 2009; Chen et al., 2021). To promote the innovative investment of time-honored brands, it is necessary to build a standardized legal system in order to guarantee the subsequent achievements and industrialization. Without a sound legal system, the market will fall into chaos. However, the “rule of man” concept in traditional Chinese culture still has a far-reaching impact on time-honored brands. For example, some time-honored brands still implement the hierarchical master-apprentice system and require absolute obedience of juniors to their superiors, which to some extent inhibit the overall innovation of the enterprises. In addition, as innovation is advocated by the market economy, continuous investment in innovation is essential if the time-honored brands aim to achieve sustainable development. However, the mentalities of adhering to past practices and keeping on the rails are deeply embedded in the traditional Chinese culture. For example, some time-honored brands still uphold the idea of being satisfied with a small wealth, which greatly hinders their innovation and development.

Based on the analysis above, we propose the following assumptions:

**Hypothesis 3a:** When traditional culture and marketization are compatible and supplementary with each other, they have complementary effects on the technological innovation of time-honored brands.

**Hypothesis 3b:** When traditional culture and marketization conflict with each other, they have mutually exclusive effects on the technological innovation of time-honored brands.

## Methodology

According to Kaya et al. (2020), the relationship between explanatory variables and explained variables is often not symmetric. Regression analysis is limited in its ability to characterize independent influences at the linear, symmetric degree level of a linear relationship, and cannot quantify asymmetric influence effects at the asymmetric degree level. However, the fsQCA method can explore the non-linear relationship between each antecedent condition and outcome from the perspective of configuration. In other words, regression analysis is used to explore the causal relationship between variables, that is, the mechanisms. However, configuration analysis is used to explore paths.

Therefore, we first use the regression analysis method to analyze the mechanism of the interaction of the dual ethical patterns on enterprise innovation from the perspective of linear symmetry. Then, the fuzzy set qualitative comparative analysis method is used to analyze the antecedent factor configuration triggering the innovation of time-honored brand enterprises from the level of an asymmetric relationship, that is, to construct the key path that triggers the innovation of time-honored brands. Thus, this manuscript adopts the method of double verification, based on the multiple regression and fuzzy set qualitative comparative analysis method, to construct the mechanism and key path of the dual ethical patterns to promote the innovation of time-honored brands.

Although previous studies have focused on the key factors influencing innovation in time-honored brands, few studies have examined the impact on innovation of time-honored firms from the dual ethical perspective formed by traditional culture and the level of marketization, and most of these studies have used a case study approach (Wang et al., 2022; Yu and Jin, 2022). However, case study methodology has the disadvantage that findings cannot be generalized. Therefore, this manuscript uses regression analysis to better explain the causal logic between the variables. Also, despite the fact that fsQCA has been used in some studies of firm innovation (Kaya et al., 2020; Huang et al., 2022), these studies have failed to explore the group effects of the interdependence and combination of traditional culture, level of marketability, and other contextual factors affecting innovation in time-honored brands. Table 1 shows the relevant previous studies on firm innovation.

**TABLE 1:** Relevant previous studies on firm innovation.

References	Description	Methods
Wang et al. (2022)	Used Zhang Xiaoquan as a case study to analyze how old companies can use new marketing and communication tools to improve their brand image and promote innovation in the new media era	Case study
Yu and Jin (2022)	Took Lidu Liquor as an example to explore whether value co-creation is helpful for achieving the innovation of time-honored brands	Case study
Wang and Wang (2020)	Analyzed the relationship between the brand growth and R&D	OLS
Kaya et al. (2020)	Used fuzzy sets (fsQCA) to explore the combined effect of organizational learning, inter-organizational communication, and collaboration for innovation on innovative performance	fsQCA
Huang et al. (2022)	Applied fsQCA method to investigate how five entrepreneurship policies (i.e., technology transfer, fiscal and tax, digital transformation, talent, and government innovation management policy) interact to influence regional innovation capability	fsQCA

Source: The authors.

### Sample selection and data sources

This study takes the “China Time-honored Brand” enterprises recognized by the Ministry of Commerce as the research object. Considering the availability of data, listed Chinese time-honored brand companies have been selected as the research samples, with a period from 2009 to 2019. The reason is that due to the impact of the world financial crisis in 2008 and the COVID-19 pandemic in 2020, the data in this period is volatile and therefore not representative or generally applicable. After excluding companies with incomplete data and without investment in innovation, and removing the influence of extreme values by winsorizing at the 1 and 99% quantiles of the sample data, we obtained a total of 343 observed values of unbalanced panel data among 42 companies. The data source of this manuscript is WIND, CSMAR, CCER database, Baidu search, etc. Among them, the innovation of time-honored brands comes from the CCER database; the control variables come from the WIND and CSMAR databases; the marketization level is calculated based on the provincial marketization index report; the data on traditional culture comes from the CSMAR database, and Baidu Map.

### The measurement approach of variables

#### Traditional culture (REL)

Drawing on the research of Kung and Ma (2014), we measured the traditional culture using the number of temples within 200 km of the registered place of the listed company. The numbers of temples within 100 and 300 km are selected as substitution variables for the robustness test.

#### Marketization degree (Ins)

This manuscript uses the total marketization index score of each region in the Marketization Index Report of Different Provinces in China (2011) compiled by Fan et al. (2011) and others to evaluate the degree of marketization in different regions. This variable takes a value of 0–10. The higher the value, the better the institutional environment in a particular region. Since the latest marketization index was published in 2018, with values available from 2008 to 2016, this manuscript draws on the practice of Wang and Ma (2019) and uses the moving average method to calculate the marketization index of each province in 2017, 2018, and 2019.

#### Innovation of time-honored brands (Tech)

Drawing on the method by Lin et al. (2017), we measured Tech using logarithm of R&D investment. Referring to the practice of Yoo and Rhee (2013), we also selected the ratio of R&D investment to total assets as a substitute variable for the robustness test.

#### Control variables

Enterprise size (Size) is measured by the natural logarithm of the total assets at the end of the period (Lin et al., 2020). The corporate property (CSP) is measured by a 0–1 variable, and the state-owned enterprise is assigned either a value of 1 or a value of 0 (Wang and Wang, 2020). Industry competition environment (HHI) is measured by the Herfindahl index (Jia et al., 2021). The larger the HHI index, the stronger the industry competitiveness. The firm growth (Grow) is measured by the growth rate of the main business (Liu and Jiang, 2016). The size of the board of directors (Id) is measured by the total number of directors (Kanakriyah, 2021). The size of the supervisory board (Bss) is determined by the total number of supervisors (Sarfraz et al., 2020). The shareholding ratio of executives (Gdr) is measured by the ratio of the total number of shares held by executives to the total number of shares (Lin et al., 2017). The return on asset ratio (Roe) is measured by the ratio of net profit to net assets (Jia et al., 2021). The profitability (Yl) is measured by the ratio of net profit to the average total assets of the company in that year (Liu and Jiang, 2016).

## Empirical results

### Descriptive statistics and Pearson correlation analysis

The results of descriptive statistical analysis and correlation coefficient analysis are shown in Table 2. The average value of innovation of time-honored brand enterprises is 16.801, indicating that the overall innovation input level of sample enterprises is in need of being enhanced; the standard deviation of traditional culture is 882.323, showing a huge difference which indicates that sample enterprises receive different degrees of impact by traditional culture. The minimum value of the marketization level is 1.501, and the maximum value is 13.342, indicating that the time-honored brands are distributed in different regions with different marketization levels. The performance of corporate properties also shows certain variations. According to the correlation coefficient of each variable, the correlation coefficient of all variables is lower than 0.6, indicating that the model used in this study does not have severe multicollinearity and is applicable to subsequent regression analysis.

**TABLE 2:** Descriptive statistics and Pearson correlation analysis.

	Tech	Rel	Lns	Size	CSP	HHI	Grow	Id	Bss	Gdr	Roe	Yl
Tech	1											
Rel	–0.007	1										
Lns	−0.104*	−0.283***	1									
Size	0.596***	−0.363***	−0.128**	1								
CSP	0.169***	−0.150***	0.080	0.284***	1							
HHI	−0.400***	−0.277***	0.166***	–0.040	0.077	1						
Grow	0.110**	0.033	–0.002	0.123**	0.047	−0.156***	1					
Id	0.052	–0.035	–0.043	0.230***	0.167***	–0.063	0.028	1				
Bss	0.077	−0.159***	−0.090*	0.196***	0.319***	0.028	–0.043	0.200***	1			
Gdr	–0.015	–0.022	0.007	−0.091*	−0.203***	–0.004	0.115**	–0.079	−0.089*	1		
Roe	0.456***	−0.251***	−0.248***	0.428***	0.209***	−0.112**	0.206***	0.171***	0.208***	–0.002	1	
Yl	0.205***	0.068	–0.064	0.0785	–0.010	−0.163***	–0.017	0.051	0.051	0.007	0.051	1
Mean	16.801	1156.297	7.285	22.421	0.641	0.218	0.132	3.394	3.851	0.616	8.965	0.455
Std.	1.780	882.323	2.795	1.195	0.480	0.162	0.265	0.997	1.216	6.770	7.471	0.534
Min	8.072	53.000	1.501	20.400	0.000	0.041	–0.602	0.000	2.000	0.000	–0.082	–1.312
Max	20.330	2910.000	13.342	25.932	1.000	0.982	2.071	9.000	9.000	42.810	0.350	9.332

*p < 0.1, **p < 0.05, ***p < 0.01.

### Analysis of regression results

A Hausman test was first performed on the model before the regression analysis. The results show rejection of the null hypothesis, so the fixed-effects model was used instead. In addition, taking the problems of heteroskedasticity and autocorrelation into consideration, this manuscript also conducts a heteroskedasticity robustness estimation on the fixed-effects model. Model 1 in Table 3 measures the relationship between traditional culture (Rel) and the innovation of time-honored brands. In this model, the coefficient of traditional culture is positive and significant at the 1% level (β = 0.003, *t* = 4.11), indicating that traditional culture has a significant positive promotion effect on the innovation of time-honored brand enterprises. Hypothesis 1a has been verified, and Hypothesis 1b has not been verified. The regression results of the control variables show that the size of the enterprise (Size), the return on equity ratio (Roe), and the profitability (Yl) are all significantly positive at the 1% level, indicating that the three indicators are all positively correlated to the innovation input of time-honored brands; the coefficients of corporate property (CSP), the size of the board of supervisors (Bss), and the ratio of senior management shareholding (Gdr) are insignificant. Therefore, their impact on the innovation of time-honored brands remains uncertain; the coefficients of industry competition environment (HHI), firm growth (Grow), and board size (Id) are negative and significant at the levels of 1, 10, and 1%, respectively, indicating that the three indicators are negatively correlated to the innovation of time-honored brands.

**TABLE 3:** Regression analysis.

Variable	Model 1 (Tech)	Model 2 (Tech)	Model 3 (Tech)
Rel	0.003***		0.001***
	(4.11)		(5.30)
Lns		0.048**	0.137***
		(1.99)	(3.73)
Rel*Lns			−0.001***
			(−4.28)
Size	0.844***	0.770***	0.863***
	(13.20)	(12.39)	(13.77)
CSP	0.204	0.155	0.340**
	(1.39)	(1.02)	(2.28)
HHI	−3.389***	−4.021***	−3.382***
	(−8.02)	(−9.70)	(−7.98)
Grow	−0.469*	−0.427*	−0.495**
	(−1.88)	(−1.68)	(−2.03)
Id	−0.239***	−0.224***	−0.195***
	(−3.60)	(−3.32)	(−2.97)
Bss	–0.067	–0.075	–0.093
	(−1.19)	(−1.31)	(−1.37)
Gdr	0.014	0.008	0.012
	(1.19)	(0.84)	(1.24)
Roe	0.598***	0.058***	0.055***
	(6.11)	(5.74)	(5.56)
Yl	0.315***	0.352***	0.300**
	(2.61)	(2.87)	(2.55)
Year	Yes	Yes	Yes
Constant	–1.563	0.289	−3.090**
	(−1.10)	(0.21)	(−2.15)
R^2^	0.584	0.568	0.607
Adj R^2^	0.572	0.555	0.592

*p < 0.1, **p < 0.05, ***p < 0.01, and *T*-value are in parentheses.

Model 2 measures the relationship between the level of marketization (Lns) and the innovation of time-honored brands. In this model, the coefficient of marketization level is positive and shows a significant correlation at the 5% level (ß = 0.048, *t* = 1.99), indicating that the higher the level of marketization, the higher the level of innovation investment of time-honored brands. Thus, Hypothesis 2 has been verified. The regression results of the control variables are basically consistent with that of model 1, which further demonstrates the rationality of the control variables selected in this study.

Model 3 adds an extended model of traditional culture (Rel), marketization level (Lns), and the interaction term (Rel*Lns) of the two. In this model, the interaction term coefficient of traditional culture and marketization level (Rel*Lns) is negative and significant at level 1%, indicating that traditional culture and the level of marketization are mutually exclusive in the technological innovation of time-honored brands. So far, Hypothesis 3b has been verified, while Hypothesis 3a has not been verified. Compared with model 2, the regression coefficient of the control variable in model 1 is significantly positive, indicating that the corporate property is positively correlated with the innovation of time-honored brands, that is, the innovation investment is higher in state-owned time-honored brands.

### Robustness test

To further verify the reliability of the model results, a second regression analysis was made by replacing traditional culture and corporate innovation. First, drawing on the research of Li and Cai (2016), this manuscript selects the number of temples within 100 km as substitution variable 1, and the number of temples within 300 km as substitution variable 2 to conduct a second regression analysis on models 1 and 3 (as shown in Table 4). The results show that the coefficient between the innovation of traditional culture and time-honored brands is still positive and significant at level 1%, while the interaction term between traditional culture and marketization is still significantly negatively correlated at level 1%. This indicates that the empirical results drawn from the above analysis have relatively high stability. Referring to the research of Yoo and Rhee (2013), and using the ratio of R&D investment to total assets as a substitution variable for the innovation of time-honored brands, this manuscript moves on to conduct a second regression analysis on the three models. As shown in Table 5, the regression results are consistent with the previous results, thus providing corroborative evidence for the above-mentioned hypothesis.

**TABLE 4:** Regression results of robust model (substituting traditional culture).

Variable	Model 1 (Rel substitution variable 1)	Model 3 (Rel substitution variable 1)	Model 1 (Rel substitution variable 2)	Model 3 (Rel substitution variable 2)
Rel	0.001***	0.004***	0.002***	0.006***
	(3.90)	(4.87)	(3.56)	(4.56)
Lns		0.121***		0.129***
		(3.56)		(3.39)
Rel*Lns		−0.003***		−0.006***
		(−3.84)		(−3.72)
Size	0.830***	0.841***	0.829***	0.843***
	(13.09)	(13.53)	(12.96)	(13.38)
CSP	0.294**	0.413***	0.202	0.306**
	(1.98)	(2.69)	(1.37)	(2.04)
HHI	−3.449***	−3.580***	−3.469***	−3.471***
	(−8.19)	(−8.51)	(−8.18)	(−8.09)
Grow	−0.488*	−0.505**	−0.465*	−0.505**
	(−1.95)	(−2.05)	(−1.85)	(−2.04)
Id	−0.253***	−0.227***	−0.238***	−0.196***
	(−3.78)	(−3.46)	(−3.56)	(−2.95)
Bss	−0.102*	−0.132**	–0.070	–0.092
	(−1.79)	(−2.34)	(−1.23)	(−1.64)
Gdr	0.011	0.013	0.011	0.011
	(1.16)	(1.37)	(1.14)	(1.17)
Roe	0.059***	0.056***	0.058***	0.054***
	(6.03)	(5.69)	(5.96)	(5.40)
Yl	0.320***	0.320***	0.328***	0.319***
	(2.65)	(2.70)	(2.71)	(2.68)
Year	Yes	Yes	Yes	Yes
Constant	–1.033	–2.130	–1.142	−2.470*
	(−0.74)	(−1.53)	(−0.80)	(−1.72)
R^2^	0.582	0.601	0.579	0.597
Adj R^2^	0.569	0.587	0.566	0.582

*p < 0.1, **p < 0.05, ***p < 0.01, and *T*-value are in parentheses.

**TABLE 5:** Regression results of robust model (substituting time-honored brands innovation).

Variable	Model 1 (Tech Substitution variable)	Model 2 (Tech Substitution variable)	Model 3 (Tech Substitution variable)
Rel	8.901*		5.740***
	(1.94)		(4.17)
Lns		0.005***	0.001***
		(3.61)	(5.22)
Rel*Lns			−6.440***
			(−4.10)
Size	−0.001***	−0.001***	−0.001***
	(−3.43)	(−4.10)	(−3.37)
CSP	–0.004	–0.008	–0.002
	(−0.52)	(−1.07)	(−0.02)
HHI	−0.014***	−0.016***	−0.015***
	(−5.81)	(−7.22)	(−6.41)
Grow	–0.002	–0.002	−0.002*
	(−1.55)	(−1.65)	(−1.80)
Id	−0.001***	−0.001***	−0.001***
	(−3.73)	(−3.70)	(−3.14)
Bss	−0.001*	−0.005*	−0.001**
	(−1.84)	(−1.73)	(−2.20)
Gdr	0.008	0.001	0.001
	(1.43)	(1.24)	(1.39)
Roe	0.000***	0.003***	0.000
	(5.57)	(6.06)	(5.53)
Yl	0.001**	0.002**	0.001**
	(2.11)	(2.35)	(2.17)
Year	Yes	Yes	Yes
Constant	0.404***	0.416***	0.312***
	(5.08)	(5.69)	(3.96)
R^2^	0.276	0.296	0.332
Adj R^2^	0.254	0.275	0.307

*p < 0.1, **p < 0.05, ***p < 0.01, and *T*-value are in parentheses.

## Extended analysis: A qualitative comparative analysis of fuzzy sets

The above empirical research has demonstrated the basic logical composition of how the traditional culture and marketization level affect the innovation of time-honored brands. However, due to the limitation of the least-squares regression analysis, it is impossible to explore the configuration effect of the influence of traditional culture, marketization level, and related situational factors on the innovation of time-honored brands. Therefore, this section will introduce the fsQCA research method to explain the complex causal mechanism of traditional culture, marketization level, and relevant situational factors that jointly affect the innovation of time-honored brands. This section aims to explain why the innovation degree varies among different time-honored brands, thereby supplementing the limitations of the above empirical research.

### Selection and calibration of variables

**FIGURE 1:**
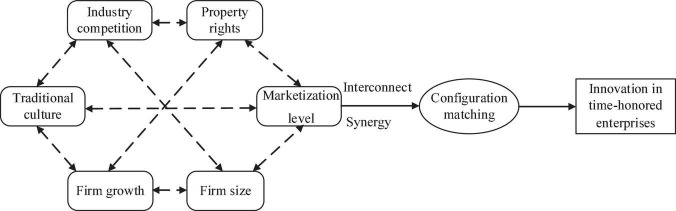
A qualitative comparative analysis of fuzzy sets (FsQCA) model.

This study selects six variables as antecedent conditions, including traditional culture, marketization level, industry competition environment, corporate property rights, enterprise growth, and enterprise size. The reasons are as follows: first, previous theoretical and empirical conclusions have provided support for the assumption that traditional culture and marketization level have an impact on the innovation of time-honored brand enterprises; second, in the extended model 3, the industry competition environment, enterprise property rights, enterprise growth, and enterprise size show significant correlations with the innovation of time-honored brands. Their direct correlations have also been verified by the existing theories (Chandrashekar, 2018; Sung, 2019). The specific model diagram is shown in Figure 1.

According to the operational rule of fsQCA, the assigned value of each variable also needs to be calibrated. Drawing on the research of Du et al. (2020), this study set the five condition variables (the corporate property right is the variable from 0 to 1 and does not need to be calibrated) and the three calibration points of one outcome variable (complete non-affiliation, intersection, and complete affiliation) as the upper quartile, median, and lower quartile, respectively. The calibration anchor points for each variable are shown in Table 6.

**TABLE 6:** Calibration anchor point of variables.

Variable		Calibrating		
		Complete non-affiliation	Intersection	Completely affiliation
Conditional variable	Rel	303.5	683.5	1,769
	Lns	5.73	7.09	9.98
	HHI	0.1	0.26	0.26
	CSP	0	/	1
	Grow	−0.02	0.08	0.16
	Size	21.77	22.86	23.83
Outcome variable	Tech	16.61	17.36	18.56

### Analysis results

#### Necessity analysis

Before conducting the configuration analysis, a condition test should be carried out. The condition test is to test whether a single condition (including logical Not and logical AND) constitutes a necessary condition for the innovation of high time-honored brands. According to the research of Morgan (2010), in the qualitative comparative analysis of fuzzy set (fsQCA), if a certain condition always exists, then the condition is the necessary condition of the result. To pass the necessary condition test, the consistency level of all antecedent variables needs to be lower than 0.9. Table 7 shows the test results of the necessary conditions for the innovation of high/non-high time-honored brands. According to the results, it can be seen that the consistency of all conditions is below 0.9. Therefore, no necessary conditions exist in the six antecedent variables for the innovation of time-honored brands. Based on the above analysis, the following sections will incorporate these six antecedent factors into the fsQCA to further explore the configurations that can boost innovation in high/non-high time-honored brands.

**TABLE 7:** Necessary condition test results.

Antecedent element	Consistency rate	Coverage rate
Traditional culture (Rel)	0.2978	0.9250
∼ Traditional culture (∼Rel)	0.0726	0.1732
Marketization level (Lns)	0.8853	0.6154
∼ Marketization level (∼Lns)	0.0924	0.3294
Industry competition (HHI)	0.0604	0.8571
∼ Industry competition (∼HHI)	0.8835	0.3895
Corporate property (CSP)	0.4849	0.6025
∼ Corporate property (∼CSP)	0.4752	0.3600
Enterprise growth (Grow)	0.1386	0.2049
∼ Enterprise growth (∼Grow)	0.1386	0.2049
Enterprise size (Size)	0.6881	0.9072
∼ Enterprise size (∼Size)	0.8845	0.6336

#### Configuration analysis

This study uses fsQCA software for configuration analysis. Drawing on the research of Morgan (2010), the consistency threshold is set to 0.8, and the acceptable number of cases is set to 1. The results are shown in Table 8. Three configuration pathways have been calculated that can boost the innovation of high time-honored brands. The three pathways have high consistency, with the consistency indicators being 0.993, 0.975, and 0.961, respectively. The overall coverage of the model is 0.830, indicating that the three configuration pathways explain the main reasons for the high innovation investment of time-honored brands.

**TABLE 8:** Acquiring the innovative configuration of high time-honored brands.

Antecedent condition	Configuration		
	**H1**	**H2**	**H3**
Rel		⊗	⊗
Lns	•	•	•
HHI	•	🌑	⊗
CSP	🌑	⊗	
Grow	•	⊗	🌑
Size	🌑	•	⊗
Consistency	0.993	0.975	0.961
Original coverage	0.296	0.320	0.177
Unique coverage	0.296	0.258	0.115
Overall consistency		0.887	
Overall coverage		0.830	

🌑 Refers to the existence of the core condition, • refers to the existence of the marginal condition, ⊗ refers to missing of the core condition, ⊗ refers to the missing of the marginal condition, and the blank means that the condition is optional.

It can be seen from Table 8 that the high marketization level appears in each of the three configuration pathways as a necessary condition. This indicates that the high level of marketization is an important condition to promote the innovation of time-honored brands. Combining specific cases, this study will make a detailed analysis of the three obtained pathways.

The antecedent configuration of H1 is Lns*HHI* CSP* Grow* Size. Its core conditions are the size of large enterprises and the nature of state-owned enterprises, and its marginal conditions are high marketization level, high industry competition environment, and high enterprise growth. This demonstrates that in an environment with fierce competition within the industry, the innovation level of large-size state-owned time-honored brands can be raised as long as these enterprises have high growth potential and a high level of marketization in the region. The influence of traditional culture has no substantial impact on their level of innovation. Typical examples of this type of configuration include Baiyun Mountain, Zhenyuantang, Phoenix, and so on. These three time-honored brands are located in the three economically developed regions of Guangdong, Zhejiang, and Shanghai. On the one hand, the high marketization level and fierce market competition in these regions force these companies to increase their efforts for research and development. On the other hand, the state-owned enterprises shoulder the important tasks of environmental protection and boosting independent innovation in China. Many preferential policies and financial support are provided by the government to encourage innovation. In addition, the increase in the main business income can also ensure the investment of innovation funds of the state-owned enterprises.

The antecedent configuration of H2 is ∼Rel* Lns* HHI*∼CSP*∼Grow* Size. The core conditions are a high industry competition environment and low enterprise growth. The marginal conditions are low traditional cultural influence, high marketization level, private enterprises, and large enterprise size. This indicates that in an environment with strong industry competition and weak influence from traditional culture, even if the private time-honored brands have weak growth motives, their innovation level can be promoted so long as they are sufficiently large in size and the marketization level of the region is high. Typical examples of this configuration include VV, Haitian, and Forever. These three time-honored brands are large size private enterprises located in the economically developed areas of Jiangsu, Guangdong, and Shanghai, where the marketization level is relatively high. Under a highly competitive environment, even when the operating income of private enterprises shrinks, they have to increase investment in research and development to meet the changing needs of consumers and achieve sustainable development.

The antecedent configuration of H3 is ∼Rel*Lns*∼HHI*Grow*∼Size. The core conditions are high enterprise growth and small enterprise size, and the marginal conditions are low traditional cultural influence, high marketization level, and low industry competition environment. This indicates that in an environment with weak industry competition and less influence by traditional culture, the innovation level of small-size time-honored brands, either state-owned or private, can be raised as long as these companies have good growth potential and a high level of marketization in the region. Typical examples of this type of configuration include Jiuzhitang, Tai’antang, Pientzehuang, etc. These three time-honored brands are all small-size companies and are located in Hunan, Guangdong, and Fujian, respectively. Compared with large enterprises, they are more flexible which beneficial for them to make faster innovative decisions according to market changes. In addition, the improvement of operating income and market mechanism also provides financial and policy support for the innovation investment of time-honored brands.

## Discussion

Innovation plays an increasingly crucial role in the competitiveness of enterprises. The development of technologies can result in dramatic changes in global social, political, and economic conditions (Ribeiro-Navarrete et al., 2021). In previous studies, many scholars have paid attention to the factors influencing corporate innovation, but they mostly analyzed from the perspective of the level of intellectual property protection (Fang et al., 2017), internal characteristics of firms such as equity structure (Muslim and Setiawan, 2021) and executive characteristics factors (Cao et al., 2022), and few studies focused on the influence of local contextual factors on corporate innovation. Even if there are studies that focus on the influence of local contextual factors on corporate innovation, there are few manuscripts that analyze time-honored brands as a research sample.

As a fusion of Chinese traditional culture and commercial culture, Chinese time-honored brands, with their unique traditional cultural background and deep cultural heritage, play a critical role in promoting China’s high-quality economic development (Zhang et al., 2021). However, as the market competition intensifies and technology iteration speeds up, it becomes increasingly difficult for time-honored brands to maintain their competitive advantage. The only way to achieve the revitalization of time-honored brands is to continuously innovate, constantly update technology, meet changing consumer needs, and strive to keep pace with the characteristics and trends of the times (Yang et al., 2021). Thus, an important question worth examining is what role traditional culture and marketization play in the innovation development of time-honored brands.

In line with previous research that concluded that traditional culture contains many ideas that inspire innovation and change (Xu et al., 2019), in our results, we found a positive relationship between traditional culture and enterprise innovation. As argued by Purkayastha et al. (2022), owing to the fact that innovation involves a high level of risk and has a long payback period, managers are reluctant to engage in innovative activities. However, the ethical value of “loyalty and trust” promoted by traditional culture can play a positive role in mitigating agency conflicts and can encourage corporate innovation (Jung et al., 2020).

Furthermore, in line with Su et al. (2021) conclusion that the market environment is a key factor influencing business innovation, we found a high degree of marketization and market competition is conducive to the innovation of time-honored brands. Similarly, Chen and Huang (2016) pointed out that a high level of marketization can promote economic structural optimization, firm competition, and technological innovation. Additionally, marketization contributes to the reduction of financing costs (Zhang et al., 2018). Marketization increases liquidity among market factors, which in turn reduces enterprises’ innovation costs and innovation risk.

However, as argued by Kaya et al. (2020), enterprise innovation is often affected by the synergy of multiple factors. Therefore, we use the fsQCA analysis method to explore the non-linear relationship between each antecedent condition and the outcome variable of technological innovation from the perspective of configuration. The results show that enterprises under little influence of traditional culture and with a high level of marketization will have a stronger potential for innovation. This indicates that in the sample enterprises, traditional culture and marketization are still in a state of incompatibility. Therefore, time-honored brands should actively carry out activities related to traditional culture, infiltrate the invisible value of traditional culture into all levels of the enterprise, and integrate various classification indicators of the marketization process to promote each other’s development. Moreover, in the context of fierce market competition, the expansion of the enterprise size is still an important force to promote the innovation of time-honored brands.

All in all, based on theoretical frameworks such as institutional theory and imprinting theory, this manuscript first reveals the theoretical logic and empirical evidence of the dual ethics of traditional culture and marketization for enterprise innovation, which not only corrects the negative cognitive bias of some scholars toward the value of traditional culture but also provides the necessary theoretical basis and policy reference for the positive role of the dual ethical patterns in realizing the strategic goal of innovation-driven development of time-honored brands.

## Conclusion

As the Chinese economy has undergone a profound transformation from a planned to a market economy, it is without a doubt that many excellent start-ups have emerged in recent years. However, time-honored brands, which have endured centuries of trials and tribulations, continue to exhibit their unique Chinese contextual imprint, and their innovative practices deserve attention as well. Nevertheless, existing research on how dual ethic patterns affect time-honored brands’ innovation activities lacks in-depth analysis and empirical research. Moreover, most of the studies on enterprise innovation have focused on linear models, ignoring interactions and the possibility that different paths could lead to the same outcome, which could be evaluated using fsQCA models. Thus, in the present study, we proposed two complementary methods, which are OLS regression and fsQCA analysis, respectively, to systematically analyze the mechanism and key path of the dual ethical patterns to promote the innovation of time-honored brands.

Regarding RQ1 (“What is the influence of traditional culture on the innovation of time-honored brands?”), we found that traditional culture is significantly positively correlated with the innovation of time-honored brands. In relation to RQ2 (“Does the difference in the level of marketization in various regions of China affect the innovation investment of time-honored brands of the locality?”), the results showed that time-honored brands located in regions with a higher level of marketization will invest more in innovation. With regard to RQ3 (“Are the two ethical patterns of traditional culture and marketization level complementary or mutually exclusive in the innovation of time-honored brands?”), we found that traditional culture and the level of marketization have mutually exclusive effects in their influence on the innovation of time-honored brands.

In addition, when it comes to fsQCA analysis, with respect to RQ4 (“How can the enterprise’s own situational variables be coordinated with the dual-ethical pattern to maximize the innovation level of time-honored brands?”), we identified three types of modes that can trigger the innovative behavior of time-honored brands. First, the configuration takes the presence of large enterprises and the nature of state-owned enterprises as core conditions leads to high innovation input. Second, the combination of high industry competition environment and low enterprise growth, supplemented by the low traditional cultural influence, high marketization level, private enterprises, and large enterprise size, are more conducive to increasing the innovation investment of the firm. Third, a configuration which includes high enterprise growth and small enterprise size as core conditions leads to high innovation investment.

In summary, we examined the question of how time-honored brands can promote their innovation activities in the local context. Our study advances time-honored brands literature by highlighting the dual ethic patterns formed by traditional culture and marketization level on the innovation investment of time-honored brands. Moreover, the study also contributes to the literature by introducing the fsQCA research method to explain the complex causal mechanism of traditional culture, marketization level, and relevant situational factors that jointly affect the innovation of time-honored brands. We hope that our results will encourage scholars to further explore the role of different cultural characteristics and market characteristics in the innovation and development of enterprises.

### Theoretical implications

Compared with the existing research, the potential theoretical contribution margin of this research is reflected in the following three aspects. First, we contribute to expanding the research paradigm of “culture and finance.” Past research works have mainly focused on the impact of culture on time-honored brands (Forêt and Mazzalovo, 2014; Chen et al., 2020; Saci et al., 2021; Zhang et al., 2021). However, this is the first study that reveals the theoretical logic and empirical evidence of the influence of traditional culture and marketization on the innovation of time-honored brands from the perspective of the dual ethical patterns. This not only enriches the research content on the innovation of time-honored brands in the local context but also provides the necessary theoretical basis for promoting the role of traditional culture as an engine to boost innovation in the process of marketization.

Second, this study provides a new research perspective and research object on the relationship between traditional culture and the process of marketization. Although the influence of traditional cultures and marketization levels on enterprises has been examined in some studies (Gu et al., 2008; Zhou et al., 2014). However, no consistent conclusions have been drawn. This study finds that traditional culture and the level of marketization have mutually exclusive effects. Furthermore, we provide empirical evidence on the debate between traditional culture and market economy by using multivariate regression analysis. In addition, this manuscript expands the research perspective on the topic of enterprise innovation. Most of the existing literature examines the macro-institutional constraints or micro-incentive mechanisms that affect enterprise innovation based on institutional logic (He and Tian, 2020; Yang et al., 2021; Huang, 2022). In this manuscript, we break through the traditional institutional theoretical framework and examine the effects and mechanisms of traditional culture on enterprise innovation behavior from an informal institutional perspective.

Third, based on the perspective of configuration, this study explores multiple paths to promote innovation investment of time-honored brands, which is a valuable revelation on how to effectively stimulate the innovation vitality of time-honored brands. Previous studies failed to explore the configuration effect of the influence of traditional culture, marketization level, and related situational factors on the innovation of time-honored brands (Yin et al., 2014; Vollmer, 2015; Shu et al., 2020; Chen et al., 2021). This means that previous scholars did not consider the interaction between influencing factors when doing time-honored brands related research. Thus, we contribute to the literature by introducing the fsQCA research method to explain the complex causal mechanism of traditional culture, marketization level, and relevant situational factors that jointly affect the innovation of time-honored brands.

### Practical implications

First, time-honored brands should realize the important role of traditional culture in their innovation, and maintain confidence in their own culture. Undoubtedly, traditional culture is the foundation of the culture of time-honored brands. Thus, time-honored brands should further incorporate the valuable essence of traditional culture to promote the innovation and development of time-honored brands. The finding that traditional culture has a “promoting effect” on the innovation of time-honored brands not only corrects the cognitive bias of some scholars on the value of traditional culture but also is conducive to enhancing cultural confidence. Moreover, it also provides a necessary theoretical basis and policy reference for carrying forward and giving play to the unique role of excellent traditional culture in guiding the innovation of time-honored brands.

Second, it goes beyond doubt that the level of marketization is an essential force in promoting the innovation of time-honored brands. Therefore, governments at all levels should continue to coordinate the promotion and improvement of the marketization process in various regions to push time-honored brands to actively carry out innovation activities. Meanwhile, traditional culture, as an implicit informal constraint and regulatory mechanism, can make up for the deficiencies of the formal system in emerging markets and motivate enterprises to invest in innovation. Therefore, in the current economic transition of China, in addition to speeding up and perfecting the construction and implementation of legal supervision and other formal systems, we should also pay attention to the construction of informal systems.

Third, according to the conclusions of this study, it can be seen that traditional culture and marketization level have mutually exclusive effects in their influencing process on the innovation of time-honored brands. Therefore, time-honored brands should choose different strategies according to their conditions, and try to avoid the degraded combination of traditional culture and the market economy, which may inhibit the innovation level of time-honored brands.

### Limitations and future research

This research also has some limitations, which also provide the direction for further research. First, this research only focuses on traditional culture and marketization level and the complex mechanism effect of four situational variables, while neglecting many other situational variables that affect the innovation of time-honored brands. Future research can be conducted to analyze the influence of the remaining situational variables. Second, Chinese traditional culture is mainly divided into three factions, namely, Confucianism, Buddhism, and Taoism. This manuscript does not respond to the question of whether different factions have a different impact on the innovation of time-honored brands. Future research can make further analysis and demonstration of this question. Third, the sample of this study only includes listed Chinese time-honored brands, while neglecting non-listed companies. Future research can discuss whether the conclusions of this study are universal and whether enterprise-type pathways exist to boost the innovation of time-honored brands.

## Data availability statement

The raw data supporting the conclusions of this article will be made available by the authors, without undue reservation, to any qualified researcher.

## Author contributions

DK: research ideas, concept and design, statistical analysis, interpretation of data, and study supervision. GL: data curation, visualization, and investigation. YJ: conceptualization, methodology, and writing—revised draft preparation. YuL: research ideas, concept and design, data curation. YiL: interpretation of analysis and writing. All authors contributed to the article and approved the submitted version.

## References

[B1] Balmer J. M. Chen W. (2017). Corporate heritage brands, augmented role identity and customer satisfaction. *Eur. J. Market.* 51 1510–1521. 10.1108/EJM-07-2017-0449

[B2] Bowonder B. Dambal A. Kumar S. Shirodkar A. (2010). Innovation strategies for creating competitive advantage. *Res. Technol. Manage.* 53 19–32. 10.1080/08956308.2010.11657628

[B3] Cai D. Shen H. Liu B. (2016). Identifying the influence of institutional factors on the R&D activities of Chinese SMEs. *Innovation* 18 54–73. 10.1080/14479338.2016.1187076

[B4] Cao X. Wang Z. Li G. Zheng Y. (2022). The impact of chief executive officers’(CEOs’) overseas experience on the corporate innovation performance of enterprises in China. *J. Innov. Knowl.* 7:100268. 10.1016/j.jik.2022.100268

[B5] Chan C. M. Makino S. Isobe T. (2010). Does subnational region matter? Foreign affiliate performance in the United States and China. *Strateg. Manage. J.* 31 1226–1243. 10.1002/smj.854

[B6] Chandrashekar D. (2018). Nature of a firm, degree of cluster linkages, and innovation: A study of Bengaluru high-tech manufacturing cluster. *Asian J. Innov. Policy* 7 103–130.

[B7] Chang S. J. Wu B. (2014). Institutional barriers and industry dynamics. *Strateg. Manage. J.* 35 1103–1123. 10.1002/smj.2152

[B8] Chen Q. Huang R. Hou B. (2020). Perceived authenticity of traditional branded restaurants (China): Impacts on perceived quality, perceived value, and behavioural intentions. *Curr. Issues Tour.* 23 2950–2971. 10.1080/13683500.2020.1776687

[B9] Chen T. Lu H. Chen R. Wu L. (2021). The impact of marketization on sustainable economic growth—evidence from West China. *Sustainability* 13:3745. 10.3390/su13073745

[B10] Chen X. Huang B. (2016). Club membership and transboundary pollution: Evidence from the European Union enlargement. *Energy Econ.* 53 230–237. 10.1016/j.eneco.2014.06.021

[B11] Du Y. Liu Q. Cheng J. (2020). What kind of ecosystem for doing business will contribute to city-level high entrepreneurial activity? A research based on institutional configurations. *Manage. World* 36 141–155.

[B12] Fan G. Wang X. Zhu H. (2011). *NERI index of marketization of China’s provinces 2011 report.* Beijing: National Economic Research Institute.

[B13] Fan Y. (2000). A classification of Chinese culture. *Cross Cult. Manage.* 7 3–10. 10.1108/13527600010797057

[B14] Fang L. H. Lerner J. Wu C. (2017). Intellectual property rights protection, ownership, and innovation: Evidence from China. *Rev. Financ. Stud.* 30 2446–2477. 10.1093/rfs/hhx023

[B15] Forêt P. Mazzalovo G. (2014). The long march of the Chinese luxury industry towards globalization: Questioning the relevance of the “China time-honored brand” label. *Luxury* 1 133–153.

[B16] Grossman G. M. Lai E. L.-C. (2004). International protection of intellectual property. *Am. Econ. Rev.* 94 1635–1653. 10.1257/0002828043052312

[B17] Gu F. F. Hung K. Tse D. K. (2008). When does guanxi matter? Issues of capitalization and its dark sides. *J. Mark.* 72 12–28. 10.1509/jmkg.72.4.012 11670861

[B18] He B. Wang J. Wang J. Wang K. (2018). The impact of government competition on regional R&D efficiency: Does legal environment matter in China’s innovation system? *Sustainability* 10:4401. 10.3390/su10124401

[B19] He J. Tian X. (2020). Institutions and innovation: A review of recent literature. *Annu. Rev. Financ. Econ.* 12 377–398. 10.1146/annurev-financial-032820-083433

[B20] Huang K. G.-L. Geng X. Wang H. (2017). Institutional regime shift in intellectual property rights and innovation strategies of firms in China. *Organ. Sci.* 28 355–377. 10.1287/orsc.2017.1117 19642375

[B21] Huang Q. (2022). Research on digital marketing intelligence innovation of Guangzhou’s Time-Honored Brands in the Era of Big Data. *Proc. Bus. Econ. Stud.* 5 40–50. 10.26689/pbes.v5i4.4200

[B22] Huang Y. Li S. Xiang X. Bu Y. Guo Y. (2022). How can the combination of entrepreneurship policies activate regional innovation capability? A comparative study of Chinese provinces based on fsQCA. *J. Innov. Knowl.* 7:100227. 10.1016/j.jik.2022.100227

[B23] Hussain S. Terziovski M. (2016). Intellectual property appropriation strategy and its impact on innovation performance. *Int. J. Innov. Manage.* 20:1650016. 10.1142/S136391961650016X

[B24] Jia L. Nam E. Chun D. (2021). Impact of Chinese government subsidies on enterprise innovation: Based on a three-dimensional perspective. *Sustainability* 13:1288. 10.3390/su13031288

[B25] Jia N. (2014). Are collective political actions and private political actions substitutes or complements? Empirical evidence from China’s private sector. *Strateg. Manage. J.* 35 292–315. 10.1002/smj.2092

[B26] Jung J. Kim S. J. Kim K. H. (2020). Sustainable marketing activities of traditional fashion market and brand loyalty. *J. Bus. Res.* 120 294–301. 10.1016/j.jbusres.2020.04.019

[B27] Kanakriyah R. (2021). The impact of board of directors’ characteristics on firm performance: A case study in Jordan. *J. Asian Finance Econ. Bus.* 8 341–350.

[B28] Kaya B. Abubakar A. M. Behravesh E. Yildiz H. Mert I. S. (2020). Antecedents of innovative performance: Findings from PLS-SEM and fuzzy sets (fsQCA). *J. Bus. Res.* 114 278–289. 10.1016/j.jbusres.2020.04.016

[B29] Keupp M. M. Beckenbauer A. Gassmann O. (2010). Enforcing intellectual property rights in weak appropriability regimes. *Manage. Int. Rev.* 50 109–130. 10.1007/s11575-009-0020-9

[B30] Klapper L. F. Love I. (2004). Corporate governance, investor protection, and performance in emerging markets. *J. Corp. Finance* 10 703–728. 10.1016/S0929-1199(03)00046-4

[B31] Kung J. K.-S. Ma C. (2014). Can cultural norms reduce conflicts? Confucianism and peasant rebellions in Qing China. *Journal of Development Economics.* 111 132–149. 10.1016/j.jdeveco.2014.08.006

[B32] Li C. Cui Z. Chen J. Zhou N. (2019). Brand revitalization of heritage enterprises for cultural sustainability in the digital era: A case study in China. *Sustainability* 11:1769. 10.3390/su11061769

[B33] Li W. Cai G. (2016). Religion and stock price crash risk: Evidence from China. *China J. Account. Res.* 9 235–250. 10.1016/j.cjar.2016.04.003

[B34] Li Y. Yang L. (2021). The neo-classical structural Functioiialism: Time-honored brands network sale and supply-side reform in China. *Int. J. Bus. Anthropol.* 11 11–21. 10.33423/ijba.v11i1.4286

[B35] Lin R. Xie Z. Hao Y. Wang J. (2020). Improving high-tech enterprise innovation in big data environment: A combinative view of internal and external governance. *Int. J. Inf. Manage.* 50 575–585. 10.1016/j.ijinfomgt.2018.11.009

[B36] Lin Z. J. Liu S. Sun F. (2017). The impact of financing constraints and agency costs on corporate R&D investment: Evidence from China. *Int. Rev. Finance* 17 3–42. 10.1111/irfi.12108

[B37] Liu X. Jiang S. (2016). Bank equity connections, intellectual property protection and enterprise innovation–A bank ownership perspective. *China J. Account. Res.* 9 207–233. 10.1016/j.cjar.2016.04.002

[B38] Marquis C. Tilcsik A. (2013). Imprinting: Toward a multilevel theory. *Acad. Manage. Ann.* 7 195–245. 10.5465/19416520.2013.766076

[B39] Marshall G. Parra A. (2019). Innovation and competition: The role of the product market. *Int. J. Ind. Organ.* 65 221–247. 10.1016/j.ijindorg.2019.04.001

[B40] Meyer J. W. Rowan B. (1977). Institutionalized organizations: Formal structure as myth and ceremony. *Am. J. Sociol.* 83 340–363. 10.1086/226550

[B41] Morgan S. L. (2010). Redesigning social inquiry: Fuzzy sets and beyond. *Soc. Forces* 88 1936–1938. 10.1353/sof.2010.0011 34409987

[B42] Murphy B. Wang R. (2006). An evaluation of stakeholder relationship marketing in China. *Asia Pac. J. Mark. Logist.* 18 7–18. 10.1108/13555850610641055

[B43] Muslim A. I. Setiawan D. (2021). Information asymmetry, ownership structure and cost of equity capital: The formation for open innovation. *J. Open Innov.* 7:48. 10.3390/joitmc7010048

[B44] Norbäck P.-J. Persson L. (2012). Entrepreneurial innovations, competition and competition policy. *Eur. Econ. Rev.* 56 488–506. 10.1016/j.euroecorev.2011.12.001

[B45] Oluwatobi S. Efobi U. Olurinola I. Alege P. (2015). Innovation in A frica: Why Institutions Matter. *South Afr. J. Econ.* 83 390–410. 10.1111/saje.12071

[B46] Pan C. Abbas J. Álvarez-Otero S. Khan H. Cai C. (2022). Interplay between corporate social responsibility and organizational green culture and their role in employees’ responsible behavior towards the environment and society. *J. Clean. Prod.* 366:132878. 10.1016/j.jclepro.2022.132878

[B47] Parra A. (2019). Sequential innovation, patent policy, and the dynamics of the replacement effect. *RAND J. Econ.* 50 568–590. 10.1111/1756-2171.12287

[B48] Piao X. Moon J. J. (2019). When does knowledge similarity help foreign firms improve performance? *Asian Bus. Manage.* 18 301–323. 10.1057/s41291-018-0048-4

[B49] Pisuke H. Kelli A. (2008). Intellectual property in an innovation-based economy. *Rev. Cent. East Eur. Law* 33 223–238. 10.1163/092598808X262614

[B50] Purkayastha A. Pattnaik C. Pathak A. A. (2022). Agency conflict in diversified business groups and performance of affiliated firms in India: Contingent effect of external constraint and internal governance. *Eur. Manage. J.* 40 283–294. 10.1016/j.emj.2021.05.004

[B51] Ribeiro-Navarrete S. Saura J. R. Palacios-Marqués D. (2021). Towards a new era of mass data collection: Assessing pandemic surveillance technologies to preserve user privacy. *Technol. Forecast. Soc. Change* 167:120681. 10.1016/j.techfore.2021.120681 33840865 PMC8019834

[B52] Saci F. Jasimuddin S. M. Hoque A. (2021). Does corporate culture matter to earnings management? Evidence from Chinese Time-honoured Brand firms. *Aust. Econ. Papers* 60 435–465. 10.1111/1467-8454.12213

[B53] Sarfraz M. Ozturk I. Shah S. G. M. Maqbool A. (2020). Contemplating the impact of the moderators agency cost and number of supervisors on corporate sustainability under the aegis of a cognitive CEO. *Front. Psychol.* 11:965. 10.3389/fpsyg.2020.00965 32536890 PMC7267056

[B54] Saura J. R. Palacios-Marqués D. Ribeiro-Soriano D. (2022). Exploring the boundaries of open innovation: Evidence from social media mining. *Technovation* 2022:102447. 10.1016/j.technovation.2021.102447

[B55] Saura J. R. Ribeiro-Soriano D. Palacios-Marqués D. (2021). From user-generated data to data-driven innovation: A research agenda to understand user privacy in digital markets. *Int. J. Inform. Manage.* 60:102331. 10.1016/j.ijinfomgt.2021.102331

[B56] Shang X. F. Choi M. C. Han F. S. Zou A. N. Kim H. E. (2021). A study on the development model of quanjude, a Time-Honored Brand. *Rev. Int. Geograph. Educ. Online* 11 2177–2185.

[B57] Sheng S. Zhou K. Z. Li J. J. Guo Z. (2018). Institutions and opportunism in buyer–supplier exchanges: The moderated mediating effects of contractual and relational governance. *J. Acad. Mark. Sci.* 46 1014–1031. 10.1007/s11747-018-0582-9

[B58] Shu P. Sun S. Yan Z. (2020). Development of time-honored brands in China: Analyzing enterprises’ value system in three senses. *Int. J. Bus. Anthropol.* 10 61–72. 10.33423/ijba.v10i2.3756

[B59] Silve F. Plekhanov A. (2018). Institutions, innovation and growth: Evidence from industry data. *Econ. Trans.* 26 335–362. 10.1111/ecot.12148

[B60] Su X. Yang X. Zhang J. Yan J. Zhao J. Shen J. (2021). Analysis of the impacts of economic growth targets and marketization on energy efficiency: Evidence from China. *Sustainability* 13:4393. 10.3390/su13084393

[B61] Sung B. (2019). Do government subsidies promote firm-level innovation? Evidence from the Korean renewable energy technology industry. *Energy Policy* 132 1333–1344. 10.1016/j.enpol.2019.03.009

[B62] Tho N. N. (2016). Confucianism and humane education in contemporary Vietnam. *Int. Commun. Chin. Cult.* 3 645–671. 10.1007/s40636-016-0076-8

[B63] Vollmer A. (2015). Conflicts in innovation and how to approach the “last mile” of conflict management research–a literature review. *Int. J. Conflict Manage.* 26 192–213. 10.1108/IJCMA-09-2012-0062

[B64] Wang H. Ma L. (2019). Ownership, corruption, and revenue regimes for infrastructure partnerships: Evidence from China. *Utilities Policy* 60:100942. 10.1016/j.jup.2019.100942

[B65] Wang L. Juslin H. (2009). The impact of Chinese culture on corporate social responsibility: The harmony approach. *J. Bus. Ethics* 88 433–451. 10.1007/s10551-009-0306-7

[B66] Wang L. Li G. Zhao W. Jin X. (2022). Research on Inheritance and Innovation of Time-honored Brands under the Background of Diversification of Media, Rise of National Tide and Consumption Upgrade–Take Zhang Xiaoquan Brand as an Example. *Front. Econ. Manage.* 3 130–137.

[B67] Wang M. Zhang Q. Wang Y. Sheng S. (2016). Governing local supplier opportunism in China: Moderating role of institutional forces. *J. Oper. Manage.* 46 84–94. 10.1016/j.jom.2016.07.001

[B68] Wang Z. Wang C. (2020). The Relationship between R&D Innovation and Brand Growth of “Time-honored Brand” Enterprises. *Manage. Rev.* 32 156–167.

[B69] Williams D. Tsiteladze D. (2019). Effectiveness of regional biotechnology clusters to support innovation activities: Case of biotech cluster in Russia. *Glob. Bus. Econ. Rev.* 21 409–426. 10.1504/GBER.2019.099392 35009967

[B70] Xia C. Zhang Z. Zhang C. (2020). When the deterrence effects of Guanxi on Opportunism can be Reinforced? A combined perspective of traditional ethics and market ethics. *J. Manage. World* 36 130–145.

[B71] Xu X. Li W. Lee W. (2019). Confucian tradition and corporate innovation: The power of culture. *J. Financ. Res.* 9 112–130.

[B72] Yang M.-J. Li N. Lorenz K. (2021). The impact of emerging market competition on innovation and business strategy: Evidence from Canada. *J. Econ. Behav. Organ.* 181 117–134. 10.1016/j.jebo.2020.10.026

[B73] Yao Y. Yueh L. (2009). Law, finance, and economic growth in China: An introduction. *World Dev.* 37 753–762. 10.1016/j.worlddev.2008.07.009

[B74] Yi L. Khan M. S. Safeer A. A. (2022). Firm innovation activities and consumer brand loyalty: A path to business sustainability in Asia. *Front. Psychol.* 13:942048. 10.3389/fpsyg.2022.942048 35959050 PMC9358993

[B75] Yin S. Lu F. Yang Y. Jing R. (2014). Organizational culture evolution: An imprinting perspective. *J. Organ. Change Manage.* 27 973–994. 10.1108/JOCM-05-2013-0080

[B76] Yoo T. Rhee M. (2013). Agency theory and the context for R&D investment: Evidence from Korea. *Asian Bus. Manage.* 12 227–252. 10.1057/abm.2013.2

[B77] Yu K. Jin M. (2022). The revival mechanism of time-honored brand from the perspective of brand authenticity and value Co-Creation: Based on Lidu Liquor Case Study. *Chin. J. Manage.* 19 486–494.

[B78] Zhang S.-N. Li Y.-Q. Liu C.-H. Ruan W.-Q. (2021). A study on China’s time-honored catering brands: Achieving new inheritance of traditional brands. *J. Retail. Consum. Serv.* 58:102290. 10.1016/j.jretconser.2020.102290

[B79] Zhang T. Yue H. Zhou J. Wang H. (2018). Technological innovation paths toward green industry in China. *Chin. J. Popul. Resour. Environ.* 16 97–108. 10.1080/10042857.2018.1475902

[B80] Zhao Y. (2022). Legal environment, technological innovation and Sustainable economic growth. *Front. Psychol.* 13:929359. 10.3389/fpsyg.2022.929359 35967692 PMC9366719

[B81] Zhou K. Z. Li J. J. Sheng S. Shao A. T. (2014). The evolving role of managerial ties and firm capabilities in an emerging economy: Evidence from China. *J. Acad. Mark. Sci.* 42 581–595. 10.1007/s11747-014-0371-z

[B82] Zhou L. Hui M. K. Zhou L. Li S. (2022). Cultural congruity and extensions of corporate heritage brands: An empirical analysis of time-honored brands in China. *J. Consum. Behav.* 21 1092–1105. 10.1002/cb.2057

